# Reactivity of a Recombinant Esterase from *Thermus thermophilus* HB27 in Aqueous and Organic Media

**DOI:** 10.3390/microorganisms10050915

**Published:** 2022-04-27

**Authors:** Roberto González-González, Pablo Fuciños, Elisa Beneventi, Olalla López-López, Begoña Pampín, Ramón Rodríguez, María Isabel González-Siso, Jacobo Cruces, María Luisa Rúa

**Affiliations:** 1 Biochemistry Laboratory, CITACA-Agri-Food Research and Transfer Cluster, Campus Auga, Universidade de Vigo, 32004 Ourense, Spain; robergg@uvigo.es (R.G.-G.); pablo.fucinos@inl.int (P.F.); 2 GalChimia, SA, Parque Empresarial de Touro, Parcelas 26-27, Fonte Díaz, 15822 Touro, Spain; elisa.beneventi@rd.nestle.com (E.B.); begona.pampin@agomab.com (B.P.); ramon.rodriguez@galchimia.com (R.R.); jacobo.cruces@galchimia.com (J.C.); 3 Grupo EXPRELA, Centro de Investigacións Científicas Avanzadas (CICA), Facultade de Ciencias, Universidade da Coruña, 15071 A Coruña, Spain; olalla.lopez.lopez@gmail.com (O.L.-L.); isabel.gsiso@udc.es (M.I.G.-S.)

**Keywords:** *Thermus thermophilus*, KLEST-3S, carboxylesterase, thermostability, enantioselectivity, interfacial activation

## Abstract

The thermoalkalophilic membrane-associated esterase E34Tt from *Thermus thermophilus* HB27 was cloned and expressed in *Kluyveromyces lactis* (KLEST-3S esterase). The recombinant enzyme was tested as a biocatalyst in aqueous and organic media. It displayed a high thermal stability and was active in the presence of 10% (*v*/*v*) organic solvents and 1% (*w*/*v*) detergents. KLEST-3S hydrolysed triglycerides of various acyl chains, which is a rare characteristic among carboxylic ester hydrolases from extreme thermophiles, with maximum activity on tributyrin. It also displayed interfacial activation towards triacetin. KLEST-3S was also tested as a biocatalyst in organic media. The esterase provided high yields for the acetylation of alcohols. In addition, KLEST-3S catalyzed the stereoselective hydrolysis of (*R*,*S*)-ibuprofen methyl ester (87% ee). Our results indicate that KLEST-3S may be a robust and efficient biocatalyst for application in industrial bioconversions.

## 1. Introduction

Modern biotechnology has a great impact on today’s industry. Biotechnology has offered tools to address the industrial processing concerns, both in terms of economic benefits and environmental impact. Biocatalysts are being used to decrease the reaction time and to improve the efficiency of industrial processes such as detergent manufacturing, food technology, and synthesis or modification of optically pure compounds. Moreover, by using enzymes, the desired products may be obtained in an environmentally friendly manner, requiring fewer chemicals and energy, and producing fewer undesirable by-products than traditional chemical catalysis [1,2,3]. An example would be the use of esterases (EC 3.1.1.1, carboxyl ester hydrolases) and lipases (EC 3.1.1.3, triacylglycerol hydrolases). Both enzymes catalyze the hydrolysis of esters, but esterases preferentially catalyze the hydrolysis of water-soluble esters with short acyl chains (<10 carbon atoms), while lipases catalyze the hydrolysis of water-insoluble long-chain esters (≥10 carbon atoms). Most lipases have a loop covering the active site, being poor catalysts in the absence of an interface. However, when the enzyme binds to a water–organic interface (e.g., an organic droplet or a micelle), the loop undergoes a conformational change, and the enzyme activity increases markedly. This process (interfacial activation) is frequently used as a criterion to distinguish between esterases and lipases [4,5].

Esterases and lipases have attracted great interest as biocatalysts for biotechnological applications, mainly because, besides their natural substrates, esterases and lipases also catalyze the enantio- and regioselective hydrolysis of a wide range of natural and synthetic esters. In addition, they show remarkable levels of activity and stability in non-aqueous environments, which facilitate the catalysis of several unnatural reactions such as esterification and transesterification. These unique properties make esterases and lipases ideal catalysts for use in organic chemical bioprocesses that use them to prepare enantiomerically pure chiral compounds and to protect and deprotect synthetic intermediates [6]. As enzyme biocatalysis has become widely used, scientists are urged to provide more efficient enzymes to replace chemical catalysts [7]. The main drawback associated with the use of most available esterases and lipases is their limited stability under the extreme conditions required in most industrial bio-manufacturing processes. Typically, industrial enzymes are sensitive to high temperature, extreme pH, or the presence of a high organic solvent concentration. The use of enzymes from extreme thermophiles (microorganisms living at temperatures above 70 °C) may be a solution for stability issues since, besides thermal stability, these enzymes frequently present resistance towards a number of physical and chemical denaturing agents, including organic solvents [8,9]. They display unique properties for several biocatalytic processes involved in food, paper, detergent, medicine, or pharmacy industries, and in the novel “white biotechnology” (biofuels, bioremediation), having increased its scientific interest in the recent decades [10,11,12,13]. Therefore, thermozymes have expanded the possibilities of industrial biocatalysis, and may represent a viable alternative to their mesophilic counterparts. Production of optically pure enantiomers has attracted much attention for both agrochemicals and drugs due to their more target-specific feature and fewer side effects than racemic mixtures. Non-steroidal anti-inflammatory drugs (NSAIDs) are mostly organic acids showing general structural features, a 2-aryl substituted propionic acid and contain a sterogenic centre. Ibuprofen is one of them and it is well known that the *S*-(+)-ibuprofen is 100 times more active than the *R*-(−)-ibuprofen [14]. Resolution of ibuprofen by stereoselective esterification using lipases as biocatalysts has been extensively studied and postulated as an interesting process [15,16] based on the stereoselective hydrolysis of ibuprofen methyl ester. However, for the harsh conditions often found in the industrial preparation of optically pure NSAIDs (high temperatures or exposure to organic solvents), thermophilic enzymes offer major advantages [17]. As a salient example, the esterase Est3 from *Sulfolobus solfataricus* P2 hydrolyses the *R*-ester of racemic ketoprofen methyl ester and displays an enantiomeric excess of 80% at 60 °C [18]. Related to ibuprofen, the thermoacidophilic lipolytic enzyme (499EST) from *Acidicaldus* sp. strain USBA-GBX-499 is reported to produce *S*-ibuprofen and *S*-naproxen from racemic samples [19]. In addition, the thermophilic esterase APE1547 from archaeon *Aeropyrum pernix* K1 shows strict stereoselectivity towards *S*-ibuprofen by enzyme-catalyzed enantioselective esterification [17].

*Thermus thermophilus* HB27 produces a 34 kDa membrane-associated esterase (E34Tt) that has been purified and characterized [20]. E34Tt contains a highly hydrophobic putative transmembrane (TM) domain spanning from the first 20–25 amino acid residues of the N-terminal sequence that could be critical for maintaining hyperthermophilic function and stability of E34Tt, as described in [20]. The TM sequence includes a signal peptide (with a cleavage site between residues 16 and 17) that drives the protein to the secretory pathway by a specific mechanism of excretion of *Thermus* cells [21]. E34Tt shows hyper-thermoalkalophilic properties, reaching an optimal temperature above 80 °C and high thermostability (half-life of 135 min at 85 °C). Unfortunately, the wild-type enzyme was poorly obtained and is mainly cell-bound; thus, the production of esterase variants (full length and without partial or complete TM) happened through heterologous expression in different surrogate mesophilic hosts, such as yeasts [22,23,24] and *E. coli* [25], some of its biochemical properties being compared in [26]. High glycosylation and a variable range of secretion to the extracellular medium in yeast systems, or a sharper drop in the optimal activity temperature in yeast than in *E. coli* systems, were some of the reported drawbacks. Nonetheless, a variant of the E34Tt enzyme cloned and expressed in *Kluyveromyces lactis* [22], named KLEST-3S, obtained by removing the putative secretion signal (∆N16) from the native protein, allowed a 50-fold increase in the production of esterase activity compared to *T. thermophilus* HB27. Regarding the biochemical properties, the recombinant enzyme was active and stable in a wide range of pH and temperature (roughly from pH 4.5 to 9 and from 35 °C to 70 °C), although optimal activity was found at pH 7.5 and 47.5 °C. KLEST-3S does not require detergent for activity or stability as is the case for E34Tt, with a strict requirement for detergent (CHAPS) above the CMC to prevent protein aggregation and loss of activity. In the absence of detergent, KLEST-3S was more thermoresistant than the wild-type enzyme: at 85 °C and pH 7.5, the half-life for inactivation (t½) of KLEST-3S was over 3.8 h [22], whereas for the wild type enzyme, it was 19 min [20]. Minor differences were observed in the substrate specificity, with both enzymes showing a esterasic behaviour with optimal activity towards p-nitrophenyl decanoate [22]. The high level of expression achieved with this system allowed us to gain insights into the catalytic properties of the KLEST-3S esterase, particularly on reactions in organic media using for the first-time substrates of commercial interest. Therefore, in this work, we studied the influence of organic solvents and the other variables that control the resolution of (*R*,*S*)-ibuprofen esters by the esterase KLEST-3S. Hydrolysis of several esters and alcohol and amine acetylation were also studied. Kinetic constants with a series of p-nitrophenyl esters were determined, and activity on triglycerides was detected for the first time as for any of the variants derived from the E34Tt enzyme. Our results indicate that KLEST-3S may be a robust and efficient biocatalyst for application in industrial bioconversions.

## 2. Materials and Methods

### 2.1. Materials

p-nitrophenyl esters such as caprylate (pNP C8), caprate (pNP C10), laurate (pNP C12), palmitate (pNP C14), triacetin (Tri-C2), tributyrin (Tri-C4), tricaproin (Tri-C6), tricaprylin (Tri-C8), tricaprin (Tri-C10) and triolein (Tri-C18:1) were from Sigma Aldrich (St. Louis, MO, USA). Also from Sigma Aldrich were detergents, CHAPS (3-[(3-cholamidopropyl)-dimethylammonio]-1-propanesulfonate), SDS (sodium dodecyl sulfate), Tween-20. (*R*,*S*)-ibuprofen acid was from Fluorochem (Hadfield, UK). All other reagents were purchased at the highest commercial quality from Sigma-Aldrich or Acros Organics (Belgium), and they were used without further purification. All solvents were of HPLC grade quality and were used as such.

### 2.2. Cloning and Production

Throughout the present work, biochemical properties of an N-terminally truncated variant of the *Thermus thermophilus* HB27 E34Tt esterase (YP_004875.1), expressed by means of the *Kluyveromyces lactis* NRRL-Y1140 yeast strain [22], were studied. The recombinant strain obtained was then named KLEST-3S (expressing the ∆N16 variant, with an estimated molecular weight of 34.3 kDa). The strain producing KLEST-3S was cultivated without pH control at 30 °C and 250 rpm in YPL (1% yeast extract (*w*/*v*), 2% peptone (*w*/*v*) and 2% lactose (*w/v*)). After cultivation for 72 h, lipolytic activity was recovered in the cell-free culture media. Post-incubated cell-free medium was concentrated by dia-ultrafiltration using tangential flow filtration (TFF) cartridges with a 10 kDa cut-off polyethersulfone membrane (Millipore Corporation, Burlington, MA, USA). When required, this concentrated post-incubated medium was newly concentrated by using an Amicon stirred ultrafiltration cell (10 KDa cut-off) (Millipore Corporation). The concentrated liquid constituted the crude enzyme solution, essentially free of contaminant proteins, with which all the experiments were carried out. Reactivity studies in organic media were conducted with dry samples of KLEST-3S obtained by freeze-drying the enzyme solution.

### 2.3. Analytical Methods

Protein was measured using the BCA protein assay (Pierce, Rockford, IL, USA) using bovine serum albumin as standard and following the manufacturer’s instructions. Lipolytic activity was routinely determined spectrophotometrically using p-nitrophenyl laurate (pNP C12) solubilized in ethanol (final concentrations were 10% (*v*/*v*) ethanol and 2.5 mM pNP C12). The reaction was conducted at pH 8.0, 40 °C and stopped after 10 min of reaction with cold 1 M Na_2_CO_3_, as described in [21], and the absorbance of 200 μL aliquots measured at 400 nm in a microplate reader FLUOstar Omega (BMG Labtech, Offenburg, Germany). The linear dependence of KLEST-3S activity over the 10 min reaction was previously established such that results could be compared among all variants of the wild-type enzyme [21,27]. Hereinafter, we refer to this method as the standard method. One activity unit was defined as the amount of enzyme that produced 1 µmol of p-nitrophenol per minute under standard assay conditions. The activities were expressed in U/mg protein. All measurements were carried out in triplicate.

### 2.4. Effect of Several Additives on Enzyme Activity and Temperature on Enzyme Activity and Stability

The effect of temperature on enzyme activity was studied in the range of 25–75 °C using 2.5 mM pNP C12 as substrate (final concentration in the assay) dissolved in 2-propanol (instead of ethanol as in the standard method). In 2-propanol, KLEST-3S activity decreased by 20% in comparison to ethanol, but the change was needed to increase the substrate solubility at the lowest assayed temperatures. From here on, the method followed the procedures as described previously in Section 2.3. Samples of KLEST-3S were diluted in 50 mM phosphate buffer (pH 7.5). Thermal stability over time was also studied at 60 and 70 °C using enzyme solution samples previously diluted in 50 mM phosphate buffer (pH 7.5). Aliquots (200 μL) were placed in PCR tubes that were closed and sealed with Teflon before being introduced into a thermostatised water bath (Multi Temp II, GE Healthcare, Danderyd, Sweden). At pre-set incubation times between 0 and 7 h, aliquots were taken out of the bath, quickly cooled down on ice and centrifuged (14,000× *g*, 15 s) (Mini Spin Plus, Eppendorf AG, Hamburg, Germany). Obtained supernatants were used to assay the residual activity with the standard method.

The effect of several detergents (SDS, Tween-20 and CHAPS) and water-miscible organic solvents (methanol, ethanol, isopropanol and dimethyl sulfoxide (DMSO)) on enzyme activity was investigated in 50 mM Tris/HCl (pH 8.0) buffer containing 40 mM CaCl_2_. Final detergents concentration was 1% (*w/v*) for SDS and CHAPS and 1% (*v*/*v*) for Tween-20. The final organic solvents concentration was 20% (*v*/*v*) (10% ethanol from the activity assay plus 10% from the added solvent). The activity was measured in triplicate under standard assay conditions using pNP C12 as substrate and 10% (*v*/*v*) ethanol at 40 °C. The enzyme activity was compared with the activity in the absence of detergents or organic solvents, which was set at 100% of relative activity.

### 2.5. Acyl Chain Length Preference

Substrate specificity towards triacylglycerols was investigated using triacetin (Tri-C2), tributyrin (Tri-C4), tricaproin (Tri-C6), tricaprylin (Tri-C8), tricaprin (Tri-C10) and triolein (Tri-C18:1). Then, 20 mM of each triacylglycerol was added to 5 mM Tris-HCl (pH 8.0) containing 40 mM CaCl_2_ and 0.5% (*w/v*) gum arabic. Mixtures were emulsified in a homogenizer (Ultraturrax T-25, IKA Labortechnik) for 7 min at 9500 rpm. Except for triacetin, all triglycerides were insoluble in the reaction media in the assay conditions. The activity was measured in a pH-stat (Methrom, Herisau, Switzerland) at 40 °C and pH 8.0, using 0.01 M NaOH as titrant [28]. One activity unit was defined as the amount of enzyme that produced 1 µmol of fatty acid per min under standard assay conditions. At least three assays of activity were performed for each substrate.

Substrate specificity was also determined with p-nitrophenyl esters (pNP esters) of various acyl chains: pNP octanoate (pNP C8), pNP decanoate (pNP C10), pNP laurate (pNP C12) and pNP myristate (pNP C14). Owing to their superior solubility in aqueous solutions, compared to triacylglycerols, different concentrations of each substrate were used to calculate the initial rate of the enzymatic hydrolysis reaction with each substrate. Stock solutions of pNP esters were prepared in isopropanol and not in ethanol as for the standard activity assay, in order to improve their solubility, particularly those with the longest acyl chains. Substrate concentrations used in the assays varied from 0.05 to 2.5 mM. The final concentration of the isopropanol in the assay was 10% (*v*/*v*). Reaction was conducted at pH 7.5 and 47 °C for 10 min, optimal conditions for KLEST-3S with p-nitrophenyl esters [22]. Other experimental conditions were as stated above for the standard assay with pNP laurate. Experimental data were fitted using the Michaelis–Menten equation for kinetic constants to be obtained by means of the GraphPad Prism 8.4.1 for Mac OS X software (GraphPad Software, San Diego, CA, USA).

### 2.6. Interfacial Activation

In order to analyse whether or not KLEST-3S activity was increased when the substrate concentration exceeded its solubility limit, the short chain triacetin was chosen as substrate. Assays were conducted in a pH-stat at 40 °C in a 5 mM Tris-HCl buffer (pH 8.0) containing 40 mM CaCl_2_ and variable amounts of triacetin (from 100 mM to 1 M). Then, 0.01 M NaOH was used as titrant. All assays were performed keeping the same stirring speed, while care was taken to avoid air bubbles formation in the reaction vessel [28]. The reaction was started with the addition of the enzyme, and at least triplicates for each substrate were performed.

### 2.7. Synthesis of (R,S) Ibuprofen Methyl- Propyl- Butyl Esters

Ibuprofen esters were synthesized in house (GalChimia, SA, Spain) by means of the following procedure: 1 eq. of (*R*,*S*)-ibuprofen acid was dissolved in the corresponding primary alcohol (methanol, n-propanol, n-butanol). After addition of 2 eq. of H_2_SO_4_ the mixture was refluxed with stirring overnight. Deionized water was added to the solution and then extracted twice with CH_2_Cl_2_. The organic layer was dried over anhydrous sodium sulphate, removed under reduced pressure and finally purified by column chromatography (silica gel; 30% (*v*/*v*) AcOEt/Hexane). Purity >99% was determined by HPLC-MS and ^1^H-NMR analysis.

### 2.8. General Procedure for the Enzymatic Hydrolysis of Esters

Racemic esters (0.136 mmol) were dissolved in the organic cosolvent (10% *v*/*v*) and added to KLEST-3S (8.5 U, determined using the standard assay using pNP C12 as described above) dissolved in 0.1 M sodium buffer phosphate pH 7.4. The reaction mixture was agitated under magnetic stirring at 40 or 65 °C. After a given reaction time, reactions were diluted with methanol, filtered and analysed by HPLC-MS.

### 2.9. General Procedure for the Enzymatic Acetylation of Alcohols and Amines

Alcohols or amines (0.2 mmol) were dissolved in the vinyl acetate used as acyl donor and added to a carousel tube containing 100 mg of CaCO_3_ and KLEST-3S dissolved in 100 mM buffer phosphate pH 7.4. Vinyl acetate: buffer ratio was 2:1. Reaction mixture was agitated under magnetic stirring. After completion, organic phase was dried off, properly diluted with MeOH and analysed by HPLC-MS.

### 2.10. HPLC-MS Analysis

The amount of ibuprofen, ibuprofen esters, alcohols, amines and corresponding acetylated products were analysed by HPLC-MS using a Luna C18 column. The eluent solution is composed of mobile phase A (Acetonitrile: MeOH = 1:1), mobile phase B (water), and mobile phase C (100 mM ammonium acetate pH 7). The flow rate was 0.30 mL/min, and the column temperature was 35 °C. Initial chromatographic conditions were 10% A, 85% B, 5% C with a 3 min hold followed by a gradient to 95% A, 0% B, 5% C with 9 min hold. The optical purity of ibuprofen was determined by HPLC using a chiral column ChiraCel OD-H 5 µm capable of separating the *S*- and *R*- enantiomers and comparing with the standard resolved enantiomer of ibuprofen. The column temperature was maintained at 25 °C during the analysis. The mobile phase consisted of isopropanol: n-Hexane: TFA in the ratio of 1:100:0.1. The flow rate was set to 1 mL/min, and the peaks were detected at 220 nm. Enantioselectivity of KLEST-3S for the hydrolysis of ibuprofen esters was evaluated and expressed as the enantiomeric excess (ee value) of the products of the reaction. Conversion was calculated as the percentage of ester hydrolysed; ee value was calculated as the difference between the molar fraction of the *R* and *S* enantiomers and expressed as percentage based on the *S*-ibuprofen ester.

## 3. Results and Discussion

Cloning, expression and production, and partial purification of the recombinant enzyme KLEST-3S here studied was conducted as described in a previous paper [22]. The specific activity of the enzyme used in this work was 40.80 ± 5 U/mg (protein concentration of 8.6 ± 0.6 mg/mL), using the standard method with pNP C12 as substrate. However, when KLEST-3S samples were prediluted in 50 mM phosphate buffer (pH 7.5) to adjust enzyme activity (thermostability at 60 and 70 °C, Arrhenius analysis and kinetic constants with p-nitrophenyl esters), we found some evidence of inhibition caused by phosphate (4-fold decrease in activity). Insight with regard to this issue needs to be prospected; however, there was interest in performing those experiments under these conditions, in order for results to compare with those previously obtained for the wild-type enzyme. An example of enzyme activity drop related to the presence of phosphate can be found in [29].

### 3.1. Effect of Temperature on Enzyme Activity and Stability

**Figure 1:**
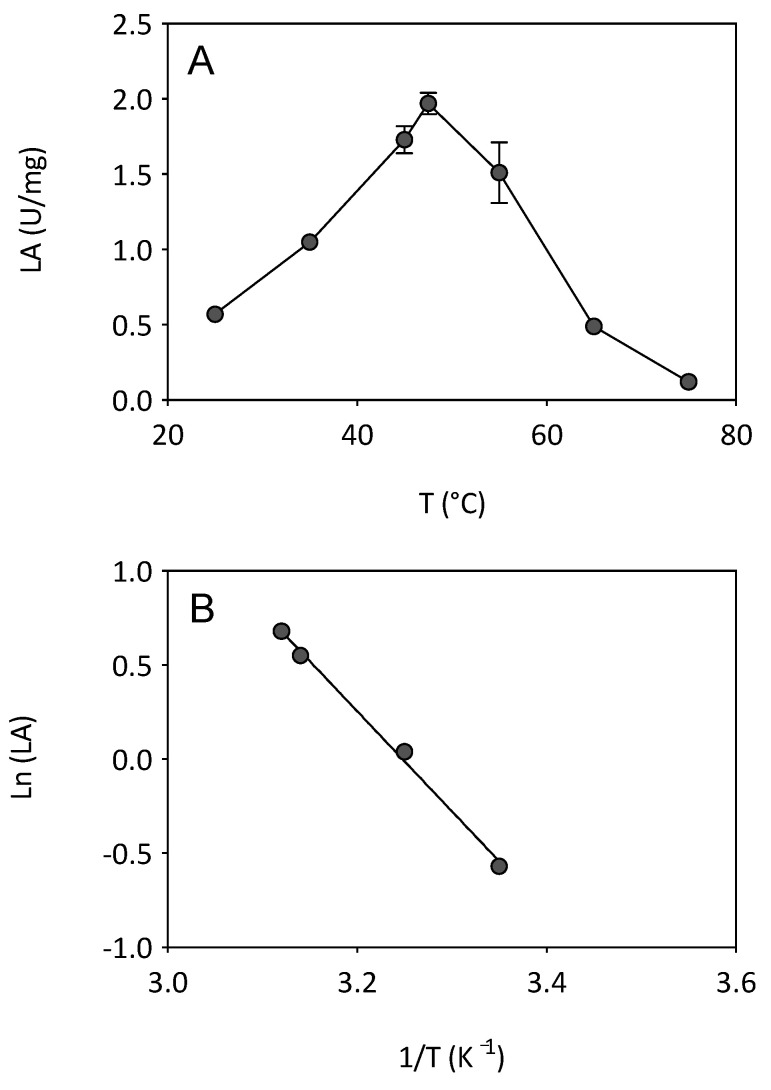
Temperature effect on the activity of KLEST-3S. (**A**) Enzymatic activities were determined with 2.5 mM pNP C12 at pH 8.0 at the different assayed temperatures. (**B**) Arrhenius plot of temperature dependence. The logarithm of the enzyme activity (units/mg) is plotted against the reciprocal of absolute temperature, T.

The relationship between activity and temperature over the range 25–80 °C was obtained using pNP C12 as substrate, as it was more stable against autohydrolysis at high temperatures when compared to esters of shorter acyl chains. KLEST-3S activity experienced abrupt changes with temperature. As reflected in Figure 1A, levels rose sharply from 25 to 50 °C and then likewise decreased sharply back down to nearly zero at 80 °C. The optimal temperature of 50 °C was almost identical to that modelled in [22] from a second-order rotatable design to determine the combined effect of pH and temperature on the KLEST-3S activity (maximum activity resulted at pH 7.5 and 47.5 °C). An Arrhenius plot was built representing the values of Ln (enzyme activity) against 1/T, where T denotes the absolute temperature in Kelvin. In the temperature range between 25 and 50 °C, the plot showed a linear relationship (Figure 1B), indicating that the enzyme activation energy (Ea) remained constant. The activation energy for the formation of the enzyme/substrate pNP C12 complex was determined from the slope (m) of this plot (m = −Ea/R, where R is the gas constant, 8.3 J/mol/K), resulting in 43.81 kJ mol^−1^ (r^2^ = 0.9959). This value is close to that of recombinant Est55 from *Geobacillus stearothermophilus* (35.7 kJ/mol) with a broader optimal range of temperature between 40 and 70 °C [30]. In addition, slightly higher than the Ea estimated for two thermophilic esterases for the hydrolysis of pNP C6; EstA3 from *Thermoanaerobacter tengcongensis* [31] and EST2 from *Bacillus acidocaldarius* [32] (36 and 31 kJ mol^−1^ for EstA3 and EST2, respectively). Although KLEST-3S shows a lower temperature for maximum activity, the good agreement indicates similar activation energy barriers for this type of enzyme and similar activation pathways associated with substrate engagement and conversion [30].

**Figure 2:**
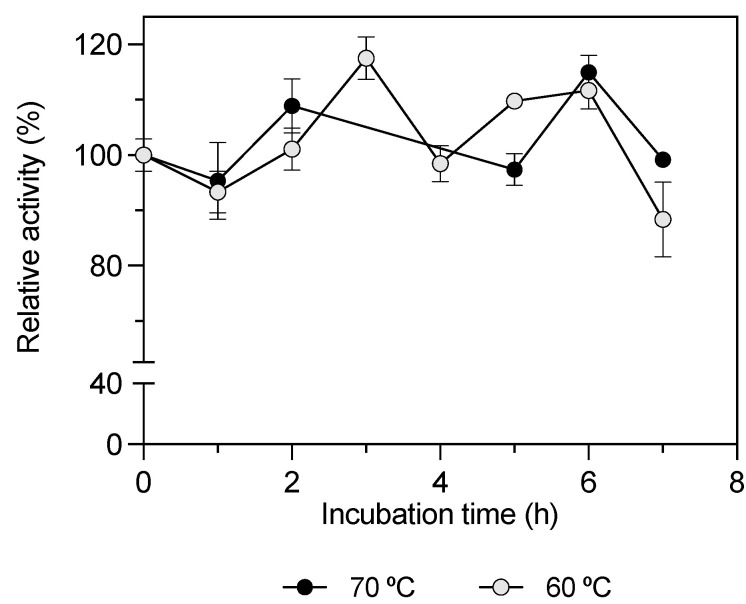
KLEST-3S thermal stability. Thermal stability profile at 60 °C (—
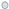
—) and 70 °C (—•—). The enzyme solution was incubated at different times between 0 and 7 h in a water bath. The pNP C12 substrate was assayed under the standard assay conditions, and residual activity was calculated; 100% KLEST-3S specific activity was 10.27 ± 0.30 U/mg.

Concerning stability, activity decay was negligible when the enzyme was incubated at 60 or 70 °C for periods of 7 h (Figure 2), which is in agreement with previous studies that showed a half-life of 230 min for this enzyme when incubated at 85 °C in the absence of micelles [22]. Having thoroughly observed optimum temperature and thermal stability data for KLEST-3S, there are several applications (e.g., the case of fine chemistry) that can benefit from highly thermostable lipolytic enzymes but need to be performed at lower temperatures. Apart from this, high stability combined with high retained activity levels at mesophilic temperatures might be of major interest to reduce costs, as in the case of industrial processes: energy savings together with extended lifetime of the biocatalysts.

### 3.2. Effect of Detergents and Solvents

The effect of organic solvents on the activity of KLEST-3S was tested within the standard activity assay, solvents being at a final concentration of 10% (*v*/*v*), and activity compared with that measured in buffer with no additives. Table 1 shows that in the presence of methanol and dimethyl sulfoxide, the activity of the recombinant KLEST-3S was respectively increased by around 25% and 4%. In the presence of ethanol, the activity decreased to almost 80%, while in the presence of isopropanol, the steepest drop was observed, being the measured residual activity of less than 22%. Organic solvents can affect enzymes in different ways; in general, low concentrations are able to potentiate the activity of many lipolytic enzymes. In contrast, high concentrations of polar organic solvents often have the opposite effect because they can favour denaturation. Thus, raised polarity levels of organic solvents are known to be more toxic for many enzymes due to stripping essential water molecules off the enzyme structure [33] or interactions with the active site modifying the essential network of hydrogen bonds for catalysis [34]. That was the case of solvents studied in this work, where methanol and dimethyl sulfoxide activated KLEST-3S, while those solvents more polar (ethanol and isopropanol) inhibited it. The effect of several surfactants (SDS, CHAPS and Tween-20) on the activity of KLEST-3S was also tested at a concentration of 1% (*w*/*v* or *v*/*v*) in the final reaction volume. In all cases, this concentration exceeded the detergents critical micellar concentration (CMC), in such a manner that the enzyme was in a micellar environment within the final reaction medium. As indicated in Table 1, detergents inhibited KLEST-3S activity to various extents. With Tween-20, activity decreased by almost 45%, while in the case of SDS and CHAPS, residual activity fell down to 38.15% and 29.02%, respectively. Stability against organic solvents is of major relevance when using enzymes for synthesis of esters. These features, along with the remarkable thermostability, make KLEST-3S attractive for many applications in industry.

### 3.3. Substrate Specificity in Aqueous Solution: Reactivity on pNP Esters and Triacylglycerols

Substrate specificity was analysed with pNP esters with acyl chain length from 8 to 14 carbon atoms. pNP esters were dissolved in 2-propanol. As indicated previously, under these conditions KLEST-3S activity decreased 20%, in comparison to ethanol, but solubility of the pNP esters with longest acyl chain (>C10) was improved, allowing for activity assays at the temperature of the assay (47.5 °C). Experimental data obtained for the hydrolysis of p-nitrophenyl esters catalyzed by KLEST-3S revealed a typical Michaelis–Menten plot, kinetic parameters being then retrieved as above explained. As summarized in Table 2, KLEST-3S shows preference for the shorter carbon chain esters studied (C8 and C10) rather than for C12 or C14, kinetics being adequately adjusted to a Michaelis–Menten model (r^2^ between 0.9907–0.999). While changes in K_M_ were small within the four p-nitrophenyl derivatives, V_max_ clearly decreased with the number of carbons within the acyl chains, being the increment more drastic between 12 and 14 carbon atoms. Thus, addition of two methylene groups to pNP C12 caused around a 10-fold decrease in V_max_. Likewise, the specificity constant (k_cat_/K_M_) showed to be two orders of magnitude lower for the C14 substrate when compared to the other three chain lengths assessed). These results pointed towards pNP C10 and pNP C8 being the most preferable substrates for KLEST-3S, as the wild-type enzyme did, and as it had already been observed for all the recombinant variants reported, regardless of the host used as expression system [20,22,25], although in those cases, the kinetic constants were not investigated, and the specificity was determined at only one substrate concentration.

**Figure 3:**
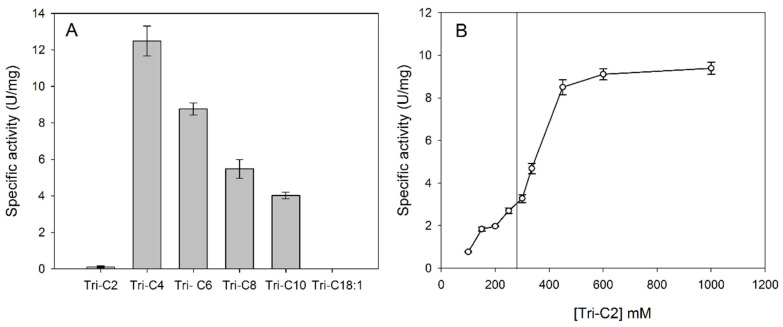
KLEST-3S substrate specificity on different triacylglycerides and kinetic profile on triacetin. (**A**) Specific activity of KLEST-3S towards several triacylglycerols. Activity was measured in pH-stat at 40 °C and pH 8.0 using emulsions of each triacylglycerol (20 mM concentration), stabilized with gum arabic. (**B**) Dependence of KLEST-3S specific activity on triacetin concentration. Activity was measured in pH-stat at 40 °C in 5 mM Tris-HCl buffer (pH 8.0) containing 40 mM CaCl_2_ and variable amounts of triacetin. The triacetin solubility limit (270 mM) is indicated by a vertical line. In both cases, data are expressed as mean values of three replicates for each substrate or triacetin concentration.

Concerning triacylglycerols, the highest specific activity of KLEST-3S, 12.49 ± 0.82 U/mg, was found towards tributyrin (short chain: C4 acyl group) (Figure 3A). With increasing chain length, activity declined progressively to reach 4.02 ± 0.18 U/mg with Tri-C10 (Figure 3A). Shorter chain length resulted in low hydrolysis levels (Tri-C2 activity was 99.12% lower than Tri-C4). No activity was detected towards the mono-unsaturated long-chain triglyceride triolein, a substrate considered typical of lipases. This latter circumstance, together with the fact that the protein showed more activity towards short chain substrates, could lead to classify it within the group of esterases.

In general, activity on esters with shorter (≤C10) and longer fatty acids (≥C10) is referred to as esterase activity and lipase activity, respectively [35,36]. Hence, according to reactivity on both, p-nitrophenyl esters and triacylglycerols, KLEST-3S should be considered an esterase rather than a lipase. Other authors rely on the so-called interfacial activation to differentiate esterases from lipases. Whereas esterases obey classical Michaelis–Menten kinetics, lipases need a minimum substrate concentration before high activity is observed, process known as interfacial activation [1,37]. The 3D-structure of many lipases has revealed that this interfacial activation is due to a hydrophobic domain (lid or flap) covering the active site when the enzyme is in the so-called closed conformation [38,39]. This lid-closed conformation maintains the catalytic triad in a hydrophobic environment isolated from aqueous solvents. Once an oil–water interface exists, the lid would pop open, providing access to the catalytic pocket [40,41,42]. Triacetin was selected to evaluate the interfacial activation of KLEST-3S [37]. These analyses were not extended to triacylglycerides with longer acyl chains, owing to their poor water solubility. Figure 3B shows how the specific activity of KLEST-3S as a function of triacetin concentration did not follow a proper Michaelis–Menten kinetics, but neither the sharp increase in activity when the solubility limit of the substrate is reached (270 mM; [28]). This kinetic behaviour does not really respond to the canonical interfacial activation that has been observed for many true lipases [43] as those produced by *Candida rugosa* lipase [40,41,44,45], CalB from *Candida antarctica* [46] or *Thermomyces lanuginose* [47], but it has been previously observed for other lipolytic enzymes such us LipC from *Pseudomonas* sp. 42A2 [48]. In addition, some lipases do not even possess a lid or do not follow interfacial kinetics, although they possess it. Even more, while it is true that interfacial activation has not been commonly reported among typical esterases, the presence of a lid or “cap”, not necessarily similar to those of true lipases, has been described in several esterases [43,49,50]. Therefore, the presence of this structure and interfacial activation should be an unsuitable criterion for classifying specific esterases. Furthermore, some authors have even suggested lipases to be pragmatically redefined as carboxylesterases acting on long-chain acylglycerols [4,43]. Using Tri-C2 as a substrate, KLEST-3S displayed a K_0.5, app_ and a V_max, app_ of 329.43 mM and 9.64 U/mg, respectively. The high K_0.5, app_ is consistent with the solubility limit of triacetin (270 mM) and is similar to that observed for *Candida rugosa* lipases [28]. Further X-ray studies of the crystal structure of KLEST-3S (yet unknown) would provide evidence that could help to understand its kinetic behaviour.

Although activity on triglycerides is much less documented in the bibliography related to esterases/lipases from thermo- and hyperthermophiles, most of the thermophilic lipolytic enzymes characterized to date possess an optimal activity with short or medium chain-length substrates (C2–C10). For example, the metagenomic esterase EstD11 shows maximum activity on pNP esters with short acyl chain (C4) [51], EstA and EstB from *Picrophilus torridus* have shown preference for C2 pNP-ester [52], p-nitrophenyl decanoate (C10) was the best substrate for EST53 from *T. maritima* [53], the esterase encoded by ORF PF2001 from *P. furiosus* exhibited the maximal specificity towards 4-methylumbelliferyl-heptanoate (C7) [54], and the highest activity of the carboxylesterases from *Sulfolobus solfataricus* P1 and *Aeropyrum pernix* K1 was towards p-nitrophenyl octanoate (C8) [55,56].

### 3.4. Biocatalytic Performance on Industrial/Benchmark Reactions

#### 3.4.1. Enantioselective Hydrolysis of Racemic Ibuprofen Esters

**Scheme 1:**
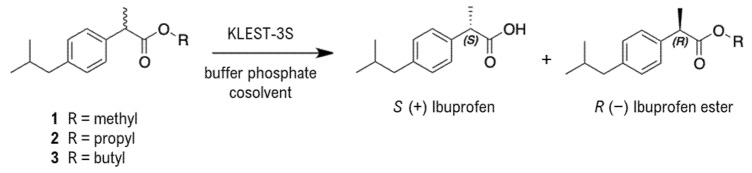
KLEST-3S-catalyzed enantioselective hydrolysis of racemic ibuprofen esters.

Non-steroidal anti-inflammatory drugs (NSAIDs) are mostly organic acids showing general structural features, a 2-aryl substituted propionic acid, and they contain a sterogenic centre. Ibuprofen is one of them, and it is well known that the S-(+)-ibuprofen is 100 times more active than the R-(−)-ibuprofen [14]. Preparation of optically pure compounds is one of the most interesting processes catalyzed by lipases. Resolution of ibuprofen by stereoselective esterification using lipases as biocatalysts has been extensively studied and postulated as an interesting process [15,16] based on the stereoselective hydrolysis of ibuprofen methyl ester. Here, we discuss the use of the new thermostable KLEST-3S to catalyze the hydrolysis of ibuprofen esters under various conditions (Scheme 1).

##### Substrate and Cosolvent Effect

**Figure 4:**
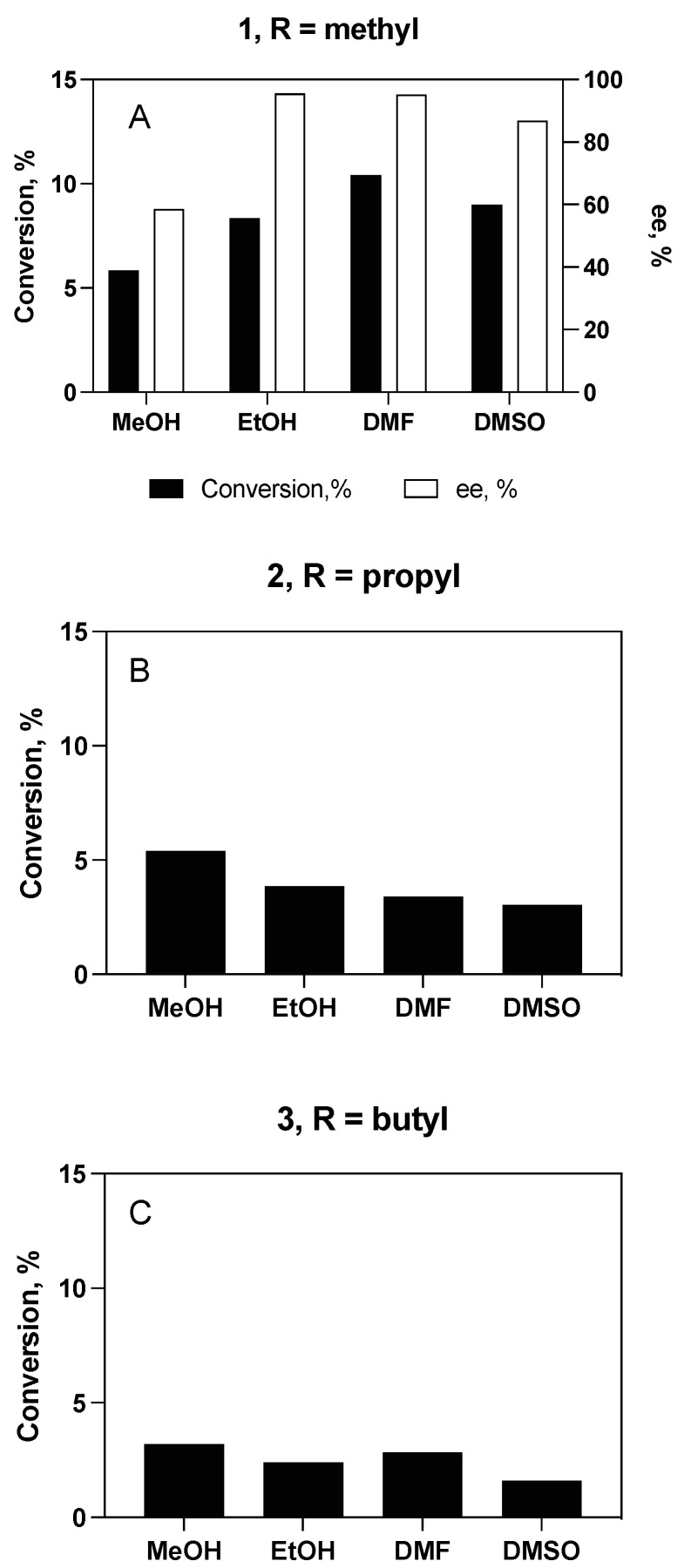
Hydrolysis of racemic ibuprofen esters to S-ibuprofen catalysed by KLEST-3S in phosphate buffer using different cosolvents. Substrates: (**A**) racemic ibuprofen methyl ester, (**B**) racemic ibuprofen propyl ester and (**C**) racemic ibuprofen butyl ester. Cosolvents: methanol (MeOH), ethanol (EtOH), dimethylformamide (DMF) and dimethylsulfoxide (DMSO). KLEST-3S, 8.5 Units (measured with pNP C12 with the standard method); reaction time, 24 h; temperature, 40 °C.

In order to evaluate the effect of alkyl chain of the substrate in terms of conversion and enantioselectivity, racemic methyl, propyl, butyl ibuprofen esters were chemically synthesized and used in the reaction system. Racemic ibuprofen methyl, propyl and butyl esters are not soluble in water; therefore, cosolvents were needed. Due to the low interfacial area available during the reaction, hydrolysis could not take place in the absence of cosolvents. To obtain a homogeneous reaction mixture, four different cosolvents were analysed for each ibuprofen ester. As seen in Figure 4, at 40 °C, ibuprofen propyl (4B) and butyl ester (4C) gave low conversion (all below 5.5%) to isolate the product for chiral analysis, while with methyl ester (Figure 4A), it was possible to isolate the acidic product and determine the ee. The best results in terms of conversion and ee were obtained using DMF (10.4% and 95,2%, respectively), which was the cosolvent selected for further investigations. These results indicate that a chain length of 1 C atom gives rise to a good substrate for KLEST-3S when it is located on the alcohol (ibuprofen esters) but on the acid moiety of the esters (pNP, TGs), showing high activity on longer acyl chains (pNP C8 or C10 and tributyrin (C4)). In similar experimental conditions (although higher temperature, 80 °C), the metagenomic EstD11 shows preference for the racemic butyl ester of ibuprofen (in 10% DMSO), reaching conversions of 10.91% and ee of 89.94% but contrary to KLEST-3S for the R-enantiomer. Other thermophilic esterase, APE1547 from archeon *Aeropyrum pernix*, K1 shows strict stereoselectivity towards *S*-ibuprofen by enzyme-catalyzed enantioselective esterification [17]. The thermoacidophilic lipolytic enzyme (499EST) from *Acidicaldus* sp. strain USBA-GBX-499 is also reported to produce *S*-ibuprofen and *S*-naproxen from racemic samples [19].

**Figure 5:**
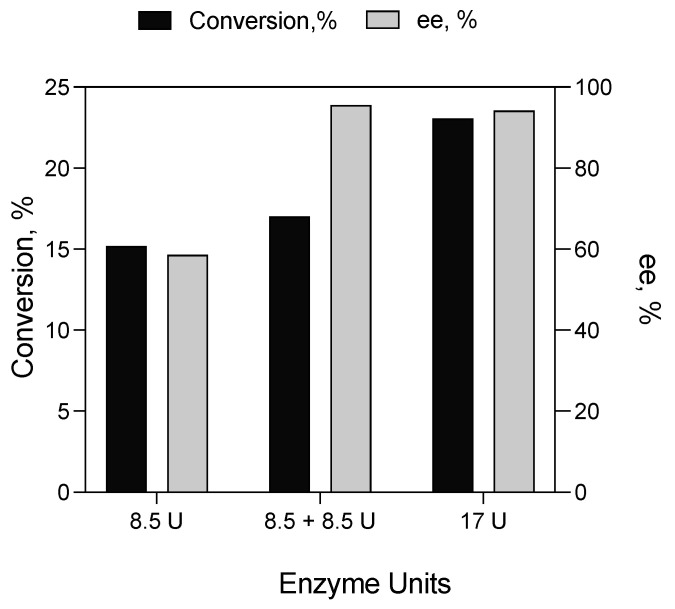
Hydrolysis of racemic ibuprofen methyl ester to *S*-ibuprofen. Reaction time, 24 h; temperature, 65 °C; cosolvent 10% (*v*/*v*) DMF. Different units of KLEST-3S and addition modes (one step or two).

The hydrolysis of racemic ibuprofen methyl ester was next studied, using DMF as cosolvent, increasing the temperature from 40 °C (Figure 4A) to 65 °C (Figure 5). By using 8.5 enzyme Units, the increase in temperature positively influenced conversion from 10.42% at 40 °C (Figure 4A) to 15.19% (Figure 5). Figure 5 also shows that feeding the enzyme during the reaction (8.5 U + 8.5 U) had little effect on conversion if compared to that obtained by adding a higher amount of enzyme (17 U) at the beginning of the reaction (17.01% conversion vs. 23.06%, respectively). However, ee was nearly the same (95.58% vs. 94.20%, respectively). Conversely, increasing the percentage of cosolvent from 10% to 20% (*v*/*v*) had a negative effect on the formation of the product and conversion dropped to nearly 5%. Hydrolysis rate was irrelevant at room temperature (not shown).

It is well known that detergents are similar to lipase substrates, and thus, they are expected to influence enzyme activity. Two different percentage of the non-ionic detergent Tween 20 (1% and 2,5%, *v*/*v*) were assayed to verify a possible positive effect on the enzyme activity, but results showed a negative effect on the enzyme, resulting in a poor reaction conversion. As shown in Table 3, with any of the concentrations tested, conversion decreased below 2% compared to 20% when the reaction took place without Tween 20.

Although most non-ionic detergents are non-denaturing [57], they can still influence esterase/lipase activity (and enantioselectivity) by a number of mechanisms, such as inducing changes in the enzyme conformation or altering the interface characteristic between the enzyme and the substrate [58]. It is also suggested that due to the long acyl ester chain of Tween 20, which has a close resemblance to esterase/lipase substrates, this detergent may act as a competitive inhibitor in the assay [59,60]. This may occur with either p-nitrophenyl laurate (Table 1) or (R,S)-ibuprofen methyl ester as substrates (Table 3).

##### Effect of Enzyme Concentration

Due to the low conversion of the reaction, different amounts of enzyme were utilized during the time and were investigated (Table 4), finding an almost linear relationship. Thus, using a four-fold amount of enzyme (8.5 U vs. 34 U), after 6 h reaction at 65 °C, the conversion to product was also quadrupled. Using 51 U of KLEST-3S (sixfold) for 24 h at the same temperature, it was possible to obtain around 30% of reaction conversion, which is the same conversion obtained, leaving the reaction for 40 h, and is the maximum value obtained for this reaction.

##### Hydrolysis of Structurally Diverse Carboxylic Esters

**Scheme 2:**
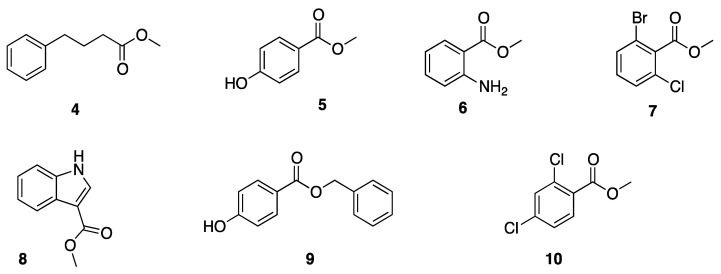
Esters used as substrate in hydrolysis reactions with KLEST-3S.

Hydrolysis of various esters (Scheme 2) was performed in the same reaction conditions used for the racemic ibuprofen methyl ester, DMF 10%, 65 °C and using 8,5 U of KLEST-3S as biocatalysts.

**Figure 6:**
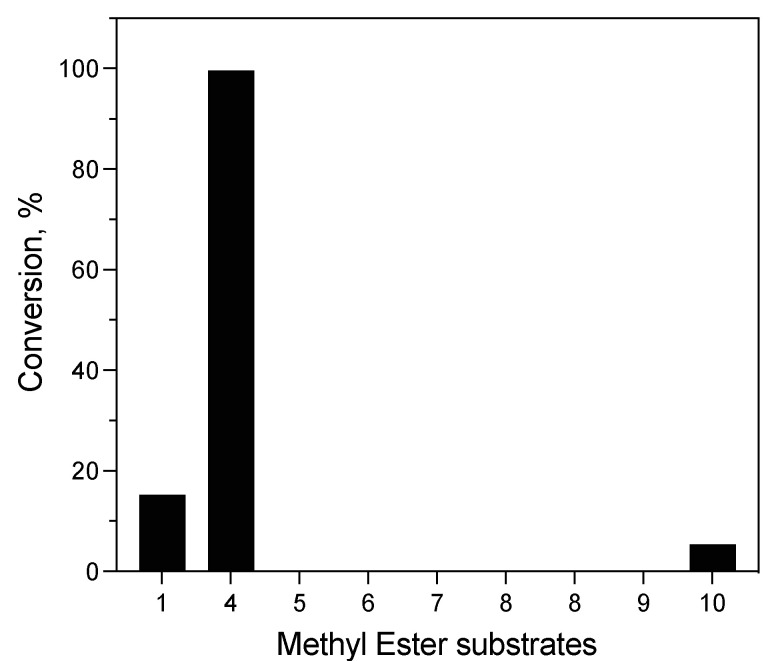
Hydrolysis of different esters to corresponding acid. Reaction time, 24 h; temperature, 65 °C; Cosolvent, 10% (*v*/*v*) DMF; KLEST-3S, 8.5 Units (measured with pNP C12 with the standard method).

As shown in Figure 6, the only positive result was obtained with the 2-phenylpropilmethyl ester (**4**) with which conversion was nearly 100% in comparison to ibuprofen methyl ester (**1**) that under the same conditions reached 15.2% conversion.

#### 3.4.2. Enzyme-Catalyzed Acetylation Reaction

**Scheme 3:**
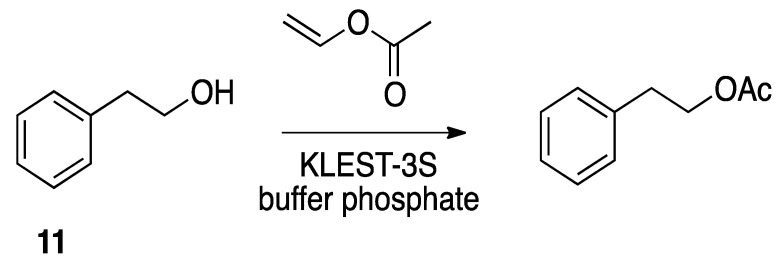
*S*-catalyzed acetylation reaction of phenylethanol.

It is well known that enzymes can be catalytically active in the presence of organic solvents. The most frequently studied lipase/esterase-catalyzed reaction in organic solvent (non-aqueous medium) is the acetylation of racemic alcohols and amines employing vinyl acetate as acyl donor. In a previous work, it was found that water is necessary for KLEST-3S activity (not shown); thereby, the reaction has been performed in a biphasic system vinyl acetate:buffer = 2:1. In such a two-phase system, the enzyme is not in direct contact with the organic solvent. Unfortunately, the presence of water in the medium allows for the competing hydrolysis reaction on the product formed and on the vinyl acetate, producing acetic acid, which strongly lowers the pH of the aqueous medium. Water content has a crucial role in this kind of reaction, and it should be removed as the major by-product of the esterification reaction. During the first tests performed, the pH issue immediately occurred. Reactions needed to be neutralized continuously with NaOH 10% in order to avoid the pH drop at 3–4, which would lead the reaction to be halted (not shown). In order to avoid the manual addition of NaOH, calcium carbonate CaCO_3_ was added to the reaction system. Calcium carbonate is nearly insoluble in water, but the little amount that is dissolved reacts with any acidic protons neutralizing them. KLEST-3S-catalyzed acetylation reaction of phenylethanol (**11**) in such water–organic solvent two-phase systems (Scheme 3) and various conditions were investigated.

**Figure 7:**
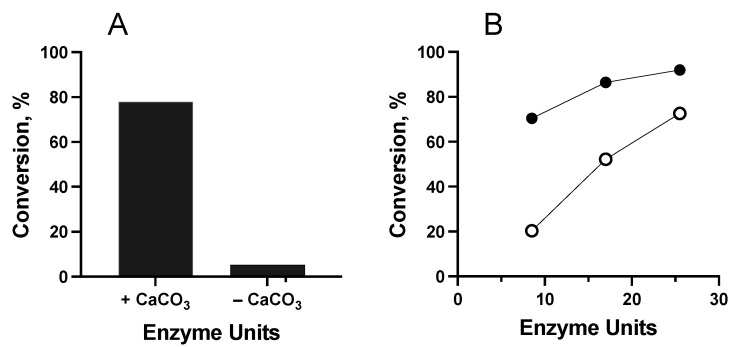
Conversion yield for the enzymatic acetylation of phenylethanol in a biphasic system. Phenylethanol, 30 mg; vinyl acetate:phosphate (2:1); temperature, 65 °C. (**A**) Conversion obtained after 24 h reaction with 8.5 U of KLEST-3S in the absence or the presence of 100 mg CaCO_3_. (**B**) Conversion yield at three enzyme concentrations (8.5, 17 and 25.5 U), after 5 h (open circles) or 24 h reaction time (closed circles).

As expected, in absence of CaCO_3_, the reaction reached 5.17% of conversion in 24 h, whereas with CaCO_3_ conversion increased to 77.95% (Figure 7A). In this condition, when the enzyme concentration in the reaction media increased, conversion also increased, as can be observed in Figure 7B, at the two reaction times considered (4 and 24 h). At 24 h, the reaction approaches almost saturation conditions already at the lowest enzyme load assayed.

**Scheme 4:**
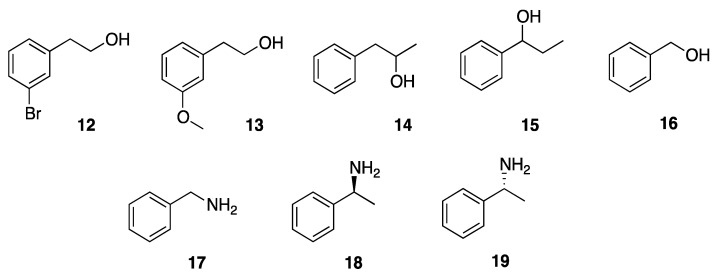
Alcohols and amines used as substrates in KLEST-3S-catalysed acetylation reaction.

Finally, a broad range of alcohols and amines was used in the same acetylation reaction (Scheme 4).

**Figure 8:**
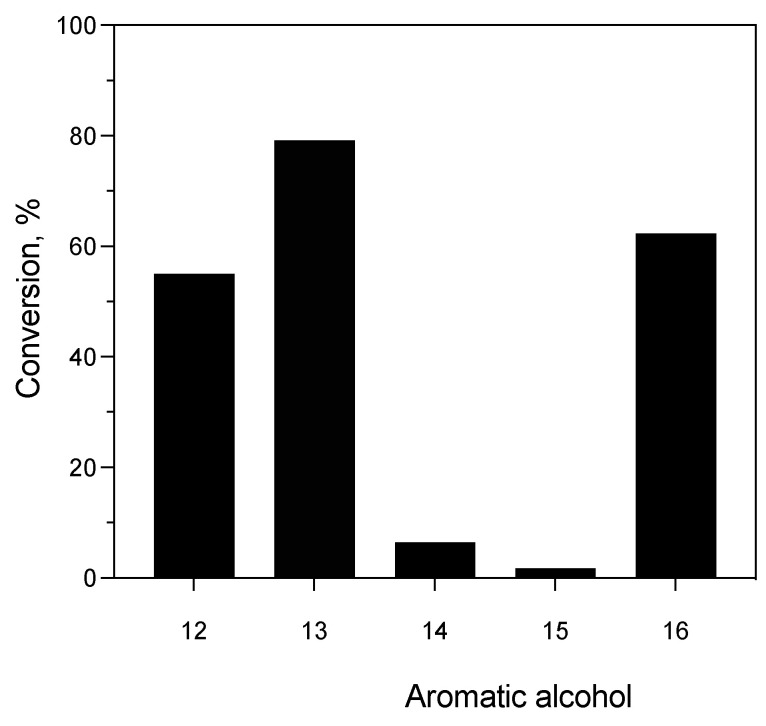
Conversion yield obtained in the acetylation reaction of different alcohols in a biphasic system vinyl acetate: buffer phosphate (2:1). KLEST-3S, 8.5 U, 24 h reaction time, 100 mg CaCO_3_, 65 °C, 30 mg of each alcohol.

Figure 8 clearly shows the preference of KLEST-3S towards primary alcohols **12**, **13** and **16**, while a drop of conversion occurs with secondary alcohols **14** and **15**, which are also the most interesting ones considering their chiral nature.

Conversely, amines were readily fully acetylated also in absence of the enzyme. This is probably due to their high reactivity at such a temperature (not shown).

## 4. Conclusions

The recombinant esterase KLEST-3S reported in this work displays high thermal activity and stability. It shows high thermoresistance, is active in solvents such as methanol or DMSO, and has high biocatalytic activity in organic–aqueous solvent mixtures indicating a great potential for bioconversions. In addition, KLEST-3S shows a peculiar property that is a rare characteristic among carboxylic ester hydrolases from extreme thermophiles, since it is active with triglycerides (maximum activity on tributyrin and no activity against triolein) and shows interfacial activation. The enzyme is inhibited by Tween 20 in both the hydrolysis of p-nitrophenyl laurate and the hydrolysis of racemic ibuprofen methyl ester, which may be a consequence of the detergent acting as a competitive inhibitor

Recombinant KLEST-3S catalyzes the stereoselective hydrolysis of racemic ibuprofen methyl ester with DMF or DMSO as organic solvent yielding the medically relevant *S*-enantiomers (87% ee in DMF). This enantiomer has better medical effects than *R*-enantiomer, which is responsible for toxic effects on the stomach. To the best of our knowledge, few thermophilic esterases have been reported to show this property. The enantioselectivity of KLEST-3S also provides high yields for the acetylation of phenylethanol with vinyl acetate as acyl donor in a biphasic system. Our results indicate that KLEST-3S may be a robust and efficient biocatalyst for application in industrial bioconversions.

## Figures and Tables

**Table 1:** Effect of different solvents and detergents on KLEST-3S activity.

**Solvent**	**Residual Activity (%)**
Control ^1^ (no additive)	100.0 ± 5.8
Methanol	125.4 ± 5.4
Dimethylsulfoxide (DMSO)	104.5 ± 5.9
Ethanol	80.9 ± 4.2
2-propanol	21.9 ± 9.3
**Detergent**	**Residual Activity (%)**
Control ^2^ (no additive)	100.0 ± 3.46
Tween 20	55.5 ± 10.5
CHAPS	21.0 ± 2.7
SDS	38.1 ± 14.4

Substrate concentration (pNP C12) was 2.5 mM; reaction time was 10 min (pH 8, 40 °C). Solvents concentration was 10% (*v*/*v*) and detergents concentration was 1% (*w*/*v*, except for Tween 20 that was *v*/*v*); 100% KLEST-3S specific activity was 37.87 ± 2.17 U/mg for Control ^1^ and 40.59 ± 1.40 U/mg for Control ^2^.

**Table 2:** Michaelis–Menten (MM) kinetic parameters retrieved for KLEST-3S activity against different chain length p-nitrophenyl esters.

Substrate	*V*_max_ (U mg^−1^)	*K*_M_ (mM)	r^2^	*K*_cat_ (s^−1^)	*k*_cat_/*K*_M_ (s^−1^ M^−1^)
pNP C8	3.57 ± 0.21	0.13 ± 0.03	0.9946	5.10 × 10^3^	3.95 × 10^7^
pNP C10	2.85 ± 0.09	0.09 ± 0.01	0.9999	4.59 × 10^3^	4.59 × 10^7^
pNP C12	2.24 ± 0.12	0.14 ± 0.03	0.9907	2.34 × 10^3^	2.34 × 10^7^
pNP C14	0.21 ± 0.01	0.19 ± 0.04	0.9948	6.05 × 10^1^	3.22 × 10^5^

Activity was measured at pH 7.5 and 47.5 °C. The substrates concentration range was from 0.05 to 2.5 mM in 10% (*v*/*v*) isopropanol (final concentrations in the assays). MW KLEST-3S, 34,330 Da.

**Table 3:** Hydrolysis of ibuprofen methyl ester to *S*-ibuprofen with KLEST-3S varying Tween 20 amounts. Enzyme units, 15. Cosolvent, 10% (*v*/*v*) DMF. Temperature 47 °C; time, 24 h.

Tween 20 (%, *v*/*v*)	Conversion (%)
0	19.9
1	1.1
2.5	1.5

**Table 4:** Hydrolysis of ibuprofen methyl ester to *S*-ibuprofen with KLEST-3S varying enzyme amount during time. Cosolvent, 10% (*v*/*v*) DMF; temperature, 65 °C.

Enzyme Units *	**Reaction Time (h)**	**Conversion (%)**
8.5	6	2.77
34	6	11.64
51	24	32.86
51	40	29.70

* Enzyme units measured with pNP C12 with the standard method.

## Data Availability

Not applicable.
